# Mapping Diversity, Equity, and Inclusion Statements in Graduate Medical Education: Protocol for a Scoping Review Across Specialties

**DOI:** 10.2196/85032

**Published:** 2026-09-10

**Authors:** Jordan Sundara, Emma Sevoian, Stephanie Wang, Linsey Golding, Vidhani Goel, Rebecca Lee, Kavita Batra

**Affiliations:** 1 Department of Medical Education, Kirk Kerkorian School of Medicine at UNLV, University of Nevada, Las Vegas, NV, United States; 2 School of Public Health, University of Nevada, Las Vegas, NV, United States; 3 Office of Research, Kirk Kerkorian School of Medicine at UNLV, University of Nevada, 1701 W Charleston Blvd, Las Vegas, NV, 89102, United States, 1 702-823-3751; 4 Obstetrics & Gynecology Residency Program, Sunrise GME Consortium, MontainView Hospital, Las Vegas, NV, United States

**Keywords:** diversity, equity, inclusion, graduate medical education, residency programs, scoping review

## Abstract

**Background:**

Persistent disparities in diversity, equity, and inclusion (DEI) remain across US residency programs despite recent accreditation requirements. In this context, DEI disparities refer to inequities in the representation, recruitment, advancement, and educational experiences of individuals from underrepresented and marginalized groups. Numerous specialty-specific studies have evaluated DEI content on residency program websites, but these studies differ in their methodologies, assessed content, and reporting practices. There is currently no comprehensive synthesis of how DEI content is presented across the different existing literature in multiple residency specialties. Therefore, a scoping review is needed to map the existing literature.

**Objective:**

This scoping review will map and summarize DEI elements reported in the peer-reviewed literature on residency program websites and related materials, identifying common practices, omissions, and opportunities for improvement.

**Methods:**

Following the population, concept, and context (PCC) framework and PRISMA-ScR (Preferred Reporting Items for Systematic Reviews and Meta-Analyses Extension for Scoping Reviews) guidelines, peer-reviewed studies published in English between January 2010 and December 2024 will be included. Eligible studies will examine quantitative, qualitative, and cross-sectional surveys; cohort studies; and program evaluations that examine DEI elements in residency programs. This includes statements, photos, departments, and curricula with elements pertaining to race; ethnicity; lesbian, gay, bisexual, transgender, queer or questioning, intersex, asexual, and other encompassing identities; disability; cultures; underrepresented in medicine populations; and religion. Searches will be conducted in the PubMed, Embase, and Scopus databases. Two reviewers will independently screen, extract, and chart data. Findings will be synthesized descriptively and presented in tables and figures.

**Results:**

As of September 2025, database searches yielded 1709 records after deduplication. Screening, data extraction, and synthesis are proceeding according to the timeline outlined in the protocol, with completion anticipated in 2026.

**Conclusions:**

This review will provide a comprehensive overview of DEI practices in residency programs across specialties, inform best practices for standardization, and support efforts to build a more equitable physician workforce.

## Introduction

Although medicine has made progress toward greater inclusivity, many medical specialties continue to lack racial and ethnic diversity. According to the Association of American Medical Colleges, in 2024, more than half of residents identified as White (53.2%), followed by Asian (30.5%) and Hispanic (10%). Orthopedic surgery and emergency medicine remain among the least diverse specialties, with more than 65% of residents identifying as White [1].

The COVID-19 pandemic brought major shifts to the residency recruitment process. In-person interviews were replaced by virtual meetings, away rotations were canceled, and facility tours were suspended. As a result, applicants increasingly relied on online resources to learn about residency programs. Although most programs maintained functional websites, many lacked the depth and quality of content necessary for applicants to make informed decisions [2]. A survey of fourth-year medical students at a single institution found that program websites were the most frequently used source of information when exploring residency programs [3]. Therefore, accurate and comprehensive website content is essential for attracting well-qualified and well-matched applicants.

Residency program websites play a central role in communicating program culture, diversity initiatives, and inclusivity. Many programs use diversity, equity, and inclusion (DEI) pages and statements for this purpose, but the presence and scope of DEI content vary considerably [4]. Wei et al [5] examined 4644 residency program websites across 26 specialties and found that only 6.2% included a diversity web page and 13.3% included a statement of commitment to diversity. Programs that highlight DEI elements tend to attract more diverse candidates, with studies showing that such programs have higher proportions of women and other groups underrepresented in medicine (URiM) compared to those that do not emphasize DEI [6,7]. Thus, residency websites represent a powerful but underused tool for attracting diverse applicants and ensuring the physician workforce better reflects the populations it serves. This review focuses specifically on residency programs in the United States, given that DEI accreditation requirements, including the Liaison Committee on Medical Education’s (LCME’s) diversity standards and the Accreditation Council for Graduate Medical Education’s (ACGME’s) DEI-related common program requirements, are governed by US-based accrediting bodies and do not directly generalize to graduate medical education systems in other countries. A diverse health care workforce is critical to building well-rounded clinical teams and improving health outcomes. Racially and ethnically diverse teams enhance patient satisfaction, strengthen patient-provider relationships, and expand access to care in underserved communities [8]. Diverse learning environments also improve engagement, better prepare trainees for complex health care settings, and reduce costs to the health system [9,10]. In the broader scientific community, ethnic diversity has been positively correlated with research impact, with studies showing that work produced by diverse teams is cited more frequently [11].

To date, several studies have examined DEI content on residency program websites, but this evidence remains fragmented across specialties. Existing work has largely consisted of single- or limited-specialty, cross-sectional analyses of website content. For example, Wei et al [5] conducted a cross-sectional analysis of 4644 program websites spanning 26 specialties and quantified the presence of diversity web pages and statements of commitment to diversity; Mallicoat et al [4] reviewed websites across obstetrics and gynecology, internal medicine, family medicine, and pediatrics for discrete DEI elements; Hayden et al [7] examined DEI elements on otolaryngology residency websites; and Chaudhry et al [12] performed a descriptive analysis within a single specialty, occupational and environmental medicine. These studies differ substantially in the specialties sampled, in the DEI elements they operationalize, and in the terminology and reporting practices they apply, and none synthesizes findings across specialties. Consequently, important gaps remain: there is no consolidated map of which DEI elements are reported, how they are defined and framed, and where coverage is concentrated or absent across the literature. This study aims to address these gaps by conducting a comprehensive scoping review of literature that examines DEI content across residency program websites in the United States. DEI content encompasses statements dedicated to diversity, including mentions of social identity groups; cultures; religions; lesbian, gay, bisexual, transgender, queer or questioning, intersex, asexual, and other encompassing identities (LGBTQIA+); disability; and URiM populations. Because residency training structures, accreditation requirements, and DEI-related policies vary substantially across countries, the present review focuses specifically on residency programs in the United States. Limiting the review to the United States allows for a more consistent evaluation of DEI content within a shared regulatory framework, particularly within the ACGME requirements related to diversity and inclusion established in 2019. The present study aims to address this gap by conducting a comprehensive scoping review of published studies that investigated DEI content across residency program websites. Our goal is to provide a consolidated reference framework that can inform best practices and guide the development of standardized, transparent, and effective DEI content for residency programs.

Recent changes in the US political landscape have altered established DEI programs within medical education [13]. In April 2025, the executive order “Reforming Accreditation to Strengthen Higher Education,” issued by President Trump, directed federal agencies to reform higher education accreditation by emphasizing student outcomes, increasing accreditor competition, and scrutinizing accreditation standards that incorporate DEI requirements, including those used in medical education [14]. While the impact of these changes in the DEI landscape has yet to be seen, these developments may influence how residency programs communicate DEI-related information to prospective applicants in the near future, further underscoring the importance of understanding the current landscape of DEI content on residency program websites. DEI initiatives increasingly draw on related antiracist and antioppressive frameworks, which explicitly attend to structural and systemic drivers of inequity rather than individual-level awareness alone. This review will identify where included studies reference such frameworks, as well as related constructs such as cultural safety, cultural humility, and cultural responsiveness, as part of their reported DEI elements.

Accordingly, the objective of this scoping review is to systematically map and characterize the DEI elements reported in the peer-reviewed literature examining residency program websites and related program materials across specialties in the United States and to describe how the presence and framing of these elements vary across disciplines. Consistent with the aims of a scoping review, we seek to chart the nature and distribution of the available evidence and to identify gaps, rather than to appraise study quality or estimate intervention effects.

The review is guided by the following question: what DEI elements are reported in the peer-reviewed literature examining residency program websites and related program materials across specialties, and how does the presence and framing of these elements vary across disciplines?

## Methods

### Ethical Considerations

Because this study does not involve human participants or patient-level data, institutional ethics approval is not required. Per PRISMA-ScR (Preferred Reporting Items for Systematic Reviews and Meta-Analyses Extension for Scoping Reviews) guidelines and to ensure methodological transparency or rigor, the protocol for this scoping review has been prospectively registered (dated July 25, 2025) on the Open Science Framework (OSF). OSF registration provides a permanent, publicly accessible record of the review protocol, helping to reduce reporting bias and enhance reproducibility.

### Inclusion and Exclusion Criteria

Eligibility criteria for this scoping review were established using the population, concept, and context (PCC) framework (Table 1). The population of interest is medical residency programs across all specialties. The central concept is DEI content and elements reported in peer-reviewed studies examining residency program websites and related program materials, defined broadly to include mission statements, recruitment strategies, mentorship initiatives, faculty diversity, and antibias training. The context is restricted to peer-reviewed studies published in English between January 2010 and December 2024. This time frame was chosen to capture the literature following key policy shifts in medical education. Specifically, the LCME adopted formal diversity standards in 2009, accompanied by the nationwide introduction of holistic review in 2009 to 2010 [15,16]. The ACGME subsequently introduced formal DEI requirements and diversity accreditation standards beginning in 2019 [17,18].

**Table 1.** Population, concept, and context (PCC) framework.

PCC components	Definition applied in this review
Population	Medical residency programs across all specialties in the United States
Concept	Diversity, equity, and inclusion, including mission or nondiscrimination statements, recruitment strategies, mentorship initiatives, faculty diversity, and antibias training
Context	Peer-reviewed, English-language studies published between January 2010 and December 2024

Eligible study designs include observational, descriptive, and survey-based studies, as well as structured website content analyses. Studies were included if they examined DEI statements, initiatives, or elements within residency programs and described or evaluated the presence, type, or impact of DEI components. Exclusion criteria were applied to conference abstracts, editorials, commentaries, letters, and reviews without primary data, as well as studies not focused on residency programs (eg, undergraduate medical education or continuing professional development) and articles not reporting DEI-related elements. Studies were further restricted to those examining residency programs based in the United States, consistent with the review’s focus on US accreditation–driven DEI standards.

### Information Sources and Search Strategy

The search strategy was developed in consultation with a health sciences librarian at the University of Nevada, Las Vegas Library (refer to the Acknowledgments section), who advised on database selection, search terminology, and efficient database searching. We did not use a maximally sensitive (high-recall) search designed to retrieve the entire body of tangentially related records, and the strategy was not independently peer reviewed using a formal instrument such as the Peer Review of Electronic Search Strategies (PRESS); given the breadth and heterogeneity of DEI-related terminology, an exhaustively sensitive search would have returned an unmanageable volume of low-relevance records without materially improving capture of the target literature. Instead, we used a deliberately broad but balanced strategy that combined controlled vocabulary (MeSH and Emtree) with free-text keywords across 4 concept blocks (graduate medical education, DEI and related constructs, the United States, and internet or website sources), joined with Boolean operators, to balance sensitivity with precision. Relevance to the review question was then ensured downstream during screening (refer to the Screening and Selection Process subsection). Literature searches will be conducted in the PubMed, Embase, and Scopus databases to identify peer-reviewed studies published between January 2010 and December 2024. Search terms will combine keywords and MeSH headings related to “diversity,” “equity,” “inclusion,” “medical residency,” and “graduate medical education.” While related constructs such as cultural safety, cultural humility, and cultural responsiveness represent conceptual evolutions beyond cultural competency, our search terms were broad and not restricted to a single construct; studies using these related terms are expected to be captured within existing search results and will be characterized during data extraction. The search strategy was developed and reported in accordance with the PRISMA-ScR guidelines [19]. A completed PRISMA-ScR checklist is provided in Checklist 1. The complete search strings for all 3 databases, including the exact Boolean combinations, field tags, and record counts, are provided in Multimedia Appendix 1, and elements of the PRISMA-S (Preferred Reporting Items for Systematic Reviews and Meta-Analyses literature search extension) searches were used to guide transparent reporting of the search.

### Screening and Selection Process

All retrieved articles will be imported into Rayyan (Rayyan Systems Inc) for screening. After deduplication, titles, abstracts, and full texts will be reviewed by 2 independent reviewers using the inclusion and exclusion criteria. Disagreements will be resolved through discussion or by a third reviewer, and if consensus cannot be reached, a third reviewer will adjudicate. To promote consistency between reviewers, a calibration exercise was conducted on a pilot sample before full screening, and relevance to the review question was ensured by screening all records against the prespecified PCC-based inclusion and exclusion criteria at both the title and abstract and full-text stages, rather than by narrowing the search itself.

### Data Extraction and Main Data Elements

Two reviewers will independently extract data into a standardized form to ensure accuracy and consistency. Extracted information will include the study title, authors, year of publication, country of study, and the residency specialty or specialties examined. Methodological characteristics such as study design and data collection approach will also be recorded. Data on the type of DEI elements reported, such as recruitment statements, mentorship programs, antibias training, or faculty demographics, will be systematically documented, along with outcomes or implications related to recruitment, representation, or residency program culture. DEI elements will be categorized into the following predefined domains for extraction: (1) statement-level elements (eg, nondiscrimination or mission statements); (2) recruitment and admissions elements (eg, holistic review practices and targeted outreach to underrepresented applicants); (3) mentorship and trainee support elements (eg, affinity groups and sponsorship programs); (4) faculty and leadership diversity elements (eg, presence of a DEI officer or committee and faculty demographic composition); (5) curricular and training elements (eg, antibias, cultural humility, or antiracism training); (6) demographic representation elements (eg, reported resident demographic data); (7) antiracist or antioppressive practice elements (eg, structural equity initiatives and explicit antiracism policies or curricula), where reported; and (8) terminology and conceptual framing used by each study (eg, cultural competency vs cultural humility, safety, or responsiveness and equity-seeking vs equity-denied language) to characterize variability in framing across the literature. This taxonomy will guide both extraction and synthesis. Each study’s key findings and reported limitations will be summarized to capture both strengths and gaps in the literature. The primary outcomes of interest are the presence, type, and variability of DEI elements within medical residency programs, as well as their reported or inferred impact on applicant recruitment, workforce diversity, and overall program inclusivity.

### Quality or Risk of Bias Assessment

Because this is a scoping review, risk of bias assessment will not be formally conducted. However, the methodological rigor and completeness of reporting in included studies and websites will be noted.

### Data Analysis and Presentation

Data from eligible peer-reviewed articles will be summarized using descriptive statistics. Frequencies and proportions will be calculated to characterize the presence and distribution of DEI elements across residency specialties. Data will be synthesized by mapping the frequency and distribution of each of the 8 predefined DEI element domains (refer to the Data Extraction and Main Data Elements subsection) across specialties and, where reported, across geographic regions and program size or type. Findings will be presented in a charting matrix cross-tabulating element domains by specialty to visualize patterns, gaps, and variability. Given the heterogeneity of study designs and outcomes, no formal meta-analysis will be performed.

## Results

**Figure 1.**
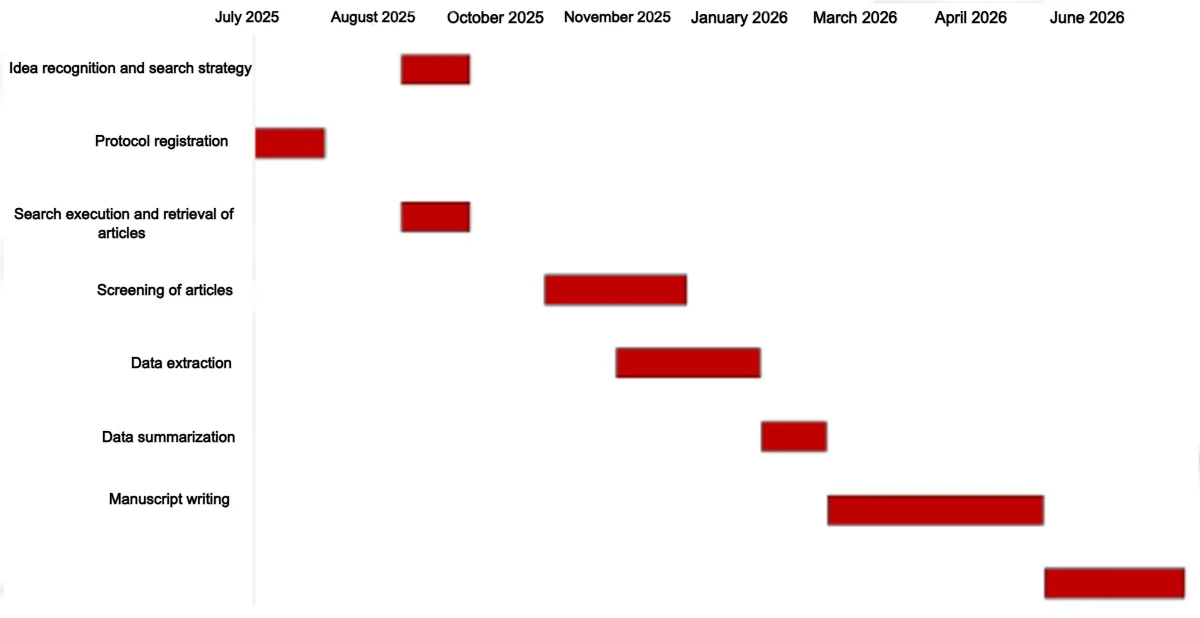
Gantt chart (timeline for review).

Database searches were completed across PubMed, Embase, and Scopus between August 2025 and September 2025 and yielded 1709 records after removal of duplicates. Title and abstract screening began in October 2025, followed by full-text review, data extraction, and charting. Data analysis and descriptive synthesis were expected to be completed in 2026. No deviations from the registered protocol occurred. The overall review timeline is summarized in Figure 1.

## Discussion

### Anticipated Findings

Our proposed scoping review is expected to provide the first comprehensive analysis of published studies that examine DEI initiatives and DEI content on US residency program websites across medical specialties. On the basis of existing literature, we anticipate large amounts of variability in the presence and scope of DEI-related content on these websites. This may include the presence or absence of DEI elements pertaining to race, ethnicity, LGBTQIA+, disability, cultures, URiM populations, and religion.

Additionally, we anticipate significant heterogeneity in how studies define, evaluate, and categorize DEI elements. Although numerous studies have examined DEI-related practices within individual specialties, current studies vary in their objectives, methodologies, and definitions of DEI, making it difficult to obtain a comprehensive understanding of how residency programs communicate DEI-related information. By mapping the existing evidence, this review will help identify which DEI domains are most commonly assessed, which specialties have been more extensively studied, and where important gaps remain.

While DEI statements can signal institutional commitment to inclusivity, prior literature suggests that their presence and content may be highly variable [12]. This study is expected to align with prior specialty-specific studies that have reported inconsistent representation of DEI content across websites. Many programs limit their statements to broad mission language, while others include specific policies, leadership roles, mentorship opportunities, or targeted resources for URiM applicants. While prior studies have described associations between DEI elements and recruitment within individual specialties, the extent to which these findings are generalizable across specialties remains unclear. By reviewing existing studies, our review hopes to provide a broader overview into how residency programs convey DEI elements to prospective trainees and to establish a centralized, accessible resource that enables applicants to more efficiently identify and compare DEI initiatives across specialties.

Overall, this scoping review aims to synthesize how DEI content is currently represented in existing literature and to identify common trends and gaps. By consolidating existing evidence, the review can help support the development of standardized frameworks that may help ensure that DEI commitments on residency websites translate into measurable progress in recruitment, retention, and ultimately health equity. In this context, our proposed scoping review may serve as a centralized resource to better compare DEI initiatives across specialties and highlight areas where more substantive efforts are needed.

### Strengths and Limitations

This scoping review applies a systematic, transparent approach guided by the PCC framework and PRISMA-ScR, ensuring rigor and reproducibility. By including peer-reviewed studies across multiple specialties and study designs, it will provide a broad synthesis of DEI elements in residency programs and identify gaps to inform best practices. Limitations include restriction to English-language, peer-reviewed publications from 2010 to 2024, which may exclude gray literature and non-English sources, as well as introduce publication bias. Variability in study design and DEI definitions may also limit comparability, and as a scoping review, it will map evidence but not assess study quality or causality. This review does not use additional a priori search terms specific to antiracist or antioppressive frameworks, cultural safety, cultural humility, or equity-denied framing; however, variability in terminology and conceptual approach used within included studies will be documented during extraction, and we identify this as a direction for more targeted future synthesis. In addition, the search strategy was developed with librarian input but was not designed to maximize sensitivity and was not formally peer reviewed using an instrument such as PRESS; while broad in scope, it prioritized a balance of sensitivity and precision, and relevant records using less common DEI-related terminology may not have been fully captured.

### Future Directions

Future research should focus on developing a standardized framework for defining and evaluating DEI content on residency program websites that would improve both consistency and meaningful comparisons across specialties and institutions. Additionally, research could explore how applicants interpret DEI-related website content and whether differences in content influence perceptions of programs.

### Dissemination

Findings from this review will be disseminated through a peer-reviewed publication and presentations at both local and national medical education conferences.

### Conclusions

The findings from this review have the potential to inform best practices for developing and standardizing DEI initiatives in graduate medical education, enhancing clarity, transparency, and accountability. A clearer understanding of the strengths and gaps in current DEI practices may also help programs align their efforts with institutional missions and accreditation expectations. Ultimately, this work aims to contribute to the broader goal of creating a more equitable and representative medical workforce. In doing so, this review may inform the development of more standardized approaches for describing and studying DEI-related website content in graduate medical education. This standardization may support clearer and more precise comparisons across different programs and specialties and facilitate future research examining DEI communication strategies.

## Supplementary material

10.2196/85032 Multimedia Appendix 1 Database search strategies.

10.2196/85032 Checklist 1 PRISMA-ScR checklist.

## Abbreviations

ACGME: Accreditation Council for Graduate Medical Education
DEI: diversity, equity, and inclusion
LCME: Liaison Committee on Medical Education
LGBTQIA+: lesbian, gay, bisexual, transgender, queer or questioning, intersex, asexual, and other encompassing identities
OSF: Open Science Framework
PCC: population, concept, and context
PRESS: Peer Review of Electronic Search Strategies
PRISMA-S: Preferred Reporting Items for Systematic Reviews and Meta-Analyses literature search extension
PRISMA-ScR: Preferred Reporting Items for Systematic Reviews and Meta-Analyses Extension for Scoping Reviews
URiM: underrepresented in medicine
