# Supplementary material for: Mapping Diversity, Equity, and Inclusion Statements in Graduate Medical Education: Protocol for a Scoping Review Across Specialties
Source: JMIR Res Protoc. 2026 Sep 10;15:e85032. doi: 10.2196/85032 (PMC13561682; doi:10.2196/85032)
Supplement: Supplementary file 2 [file resprot-v15-e85032-s001.docx]

**Multimedia Appendix 1. Database Search Strategies**

The complete PubMed, Embase, and Scopus search strategies (search numbers, full query strings, record counts, and dates) are provided below and were relocated here from the Methods for concise presentation. This appendix is also uploaded as a separate supplementary file.

Pubmed With keywords:

| Search | Query | Records Received | Date |
| --- | --- | --- | --- |
| 1 | (("Education, Medical, Graduate"[Mesh]) OR "Internship and Residency"[Mesh]) OR "medical education" OR "graduate medical education" OR residency OR "medical resident" OR "postgraduate training" OR "continuing medical education" OR "residency program" OR "medical residency program" OR internship OR "residency applicants" OR "residency application" | 743,629 | 9/14 |
| 2 | (("Cultural Diversity"[Mesh]) OR "Diversity, Equity, Inclusion"[Mesh]) OR "Cultural Competency"[Mesh] OR diversity OR diverse OR equity OR inclusion OR DEI OR "underrepresented in medicine" OR URIM OR URM | 1,485,171 |  |
| 3 | #1 AND #2 | 50,411 |  |
| 4 | ("United States"[Mesh]) OR "Population Groups, US"[Mesh] OR "USA" OR "US" OR "United States" OR "United States of America" | 9,088,764 |  |
| 5 | #3 AND #4 | 23, 831 |  |
| 6 | "Internet"[Mesh] OR internet OR website OR "web site" OR webpage | 219,164 |  |
| 7 | #5 AND #6 | 845 |  |

**Embase**

| Search | Query | Records Received | Date |
| --- | --- | --- | --- |
| 1 | 'medical education'/exp OR 'graduate medical education'/exp OR 'residency education'/exp OR 'medical education' OR residency OR 'postgraduate training' OR 'continuing medical education' OR 'residency program' OR 'medical residency program' OR internship OR 'residency applicants' OR 'residency application' | 612,674 | 9/15 |
| 2 | 'diversity, equity and inclusion'/exp OR 'cultural diversity'/exp OR 'cultural competence'/exp OR 'underrepresented minority'/exp OR diversity OR diverse OR equity OR inclusion OR DEI OR "underrepresented in medicine" OR URIM OR URM | 1,817,016 |  |
| 3 | #1 AND #2 | 32,484 |  |
| 4 | 'united states'/exp OR 'american'/exp OR usa OR us OR 'united states' OR 'united states of america' | 23,221,798 |  |
| 5 | #3 AND #4 | 21,528 |  |
| 6 | 'internet'/exp OR 'website'/exp OR internet OR 'web site' OR webpage OR website | 778,843 |  |
| 7 | #5 AND #6 | 1,025 |  |

**Scopus**

| Search | Query | Records Received | Date |
| --- | --- | --- | --- |
| 1 | TITLE-ABS-KEY ( "medical education" OR "graduate medical education" OR residency OR "medical resident" OR "postgraduate training" OR "continuing medical education" OR "residency program" OR "medical residency program" OR internship OR "residency applicants" OR "residency application" ) | 406,775 | 9/15 |
| 2 | TITLE-ABS-KEY ( "cultural diversity" OR "diversity, equity, inclusion" OR "cultural competency" OR diversity OR diverse OR equity OR inclusion OR DEI OR "underrepresented in medicine" OR URIM OR URM ) | 3,289,480 |  |
| 3 | #1 AND #2 | 20,522 |  |
| 4 | TITLE-ABS-KEY ( "United States" OR "USA" OR "US" OR "United States of America" ) | 4,264,895 |  |
| 5 | #3 AND #4 | 5,077 |  |
| 6 | TITLE-ABS-KEY ( internet OR WEBSITE OR "web site" OR webpage ) | 1,105,207 |  |
| 7 | #5 AND #6 | 334 |  |
